# Electron Microscopy in Rat Brain Slices Reveals Rapid Accumulation of Cisplatin on Ribosomes and Other Cellular Components Only in Glia

**DOI:** 10.1155/2014/174039

**Published:** 2014-12-28

**Authors:** Lidia Zueva, Yomarie Rivera, Lilia Kucheryavykh, Serguei N. Skatchkov, Misty J. Eaton, Priscila Sanabria, Mikhail Inyushin

**Affiliations:** ^1^Department of Neuroscience, Universidad Central del Caribe, Bayamón, PR 00960, USA; ^2^Department of Physiology, Universidad Central del Caribe, Bayamón, PR 00960, USA; ^3^Department of Biochemistry, Universidad Central del Caribe, Bayamón, PR 00960, USA

## Abstract

Cisplatin is a widely used, effective anticancer drug. Its use, however, is associated with several side effects including nephrotoxicity and neurotoxicity. It is known that cisplatin is accumulated in cells by the organic cation transport system and reacts with nucleotides, damaging them, but the precise target of cisplatin-induced neurotoxicity remains obscure. Here we report direct visualization of cisplatin inside brain cells using *in vivo* “cisplatin staining,” a technique that takes advantage of the high electron density of cisplatin, which contains platinum (atomic mass = 195). After applying 0.1% cisplatin to living brain slices for 30 min, we fixed the tissue and observed the accumulated cisplatin using electron microscopy. We found that cisplatin was localized mainly to ribosomes associated with endoplasmic reticulum (EPR) in glial cells and to the myelin sheath formed by oligodendrocytes around neuronal axons. Staining of nuclear DNA was moderate. Our *in vivo* “cisplatin staining” method validated that the main target of cisplatin is a direct attack on myelin and the RNA contained in ribosomes.

## 1. Introduction

Cisplatin (*cis-*diamminedichloroplatinum) (other names—Platinol AQ or Platinol) is an effective anticancer drug and one of the most commonly used chemotherapies in the USA [1]. It is transported into cancerous cells by organic cation transporters (OCTs and OCTNs; [SLC22A1–5]) [2, 3] leading to intracellular accumulation of cisplatin. The presence of OCTs was confirmed in different cancer cell lines [4, 5]. Moreover, we previously reported effective uptake of OCT substrates by glioma cells [6]. It is likely that specific uptake of cisplatin is associated with the same transport mechanism. OCTs are present in different normal tissues as well; the most well-defined examples are tubular epithelium cells in kidney [7] and astrocytes, pericytes, and oligodendrocytes in the nervous system [8, 9]. This may be the reason why cisplatin treatment is associated with numerous side effects such as nephrotoxicity and neurotoxicity [10]. While there are methods to reduce kidney damage, neurotoxicity remains a major dose-limiting factor for cisplatin therapy [11]. Patients with cisplatin-induced peripheral neuropathy typically present distal sensory ataxia, degeneration of large myelinated axons with signs of segmental demyelination and remyelination [12]. The histologic approach revealed that large axons are more frequently affected than the small ones, and nonmyelinated axons are unaffected [13]. Motor fibers are usually unaffected and overall damage to cells protected by the blood-brain barrier is less pronounced. Nevertheless, some individuals develop ototoxicity and focal encephalopathy (cortical blindness, aphasia, and focal seizures) [14, 15].

It has been generally accepted that the antineoplastic activity of cisplatin is due to the formation of platinated adducts in the nuclear DNA [16]. Adduct formation produces lesions in the nuclear DNA that lead to impaired replication and transcription and may trigger apoptosis [17]. On the other hand, it was shown that RNA can be the main target with 20-fold more platinated adduct accumulation in total cellular RNA than in DNA [18]. It was suggested that nuclear events may not be critical for the initiation of cisplatin-induced cytotoxicity [19]. Instead, it has been attributed to endoplasmic reticulum stress [20]. To obtain a more detailed understanding of the mechanisms of cisplatin-induced neurotoxicity, we used a novel sensitive method to visualize cisplatin accumulation inside living cells. Our methodology takes advantage of the high electron density of cisplatin, which contains platinum (atomic mass = 195), and allows* in vivo* “cisplatin staining” for electron microscopic assessment. A similar principle was used to show the absence of cisplatin vesicular transport in carcinoma cells [21]. The aim of the current study was to identify which organelles contain elevated levels of accumulated cisplatin products in different types of brain cells after acute application of cisplatin.

## 2. Methods

### 2.1. Animals and Slice Preparation

All experimental procedures were performed in accordance with the US Public Health Service Publication Guide for the Care and Use of Laboratory Animals and were approved by the Animal Care and Use Committee at Universidad Central del Caribe. Sprague-Dawley rats of either sex between 20 and 30 days of age were decapitated. Hippocampal slices (200 *μ*m) were prepared using a vibratome (VT1000S, Leica Microsystems GmbH, Wetzlar, Germany) containing artificial cerebrospinal fluid (ACSF) composed of (in mM) 127 NaCl, 2.5 KCl, 1.25 NaH_2_PO_4_, 25 NaHCO_3_, 2 CaCl_2_, 1 MgCl_2_, and 25 D-glucose, ice-cold, saturated with a 95% O_2_-5% CO_2_ gas mixture at pH = 7.4.

### 2.2. Application of Cisplatin

Cisplatin was added to the ACSF to final concentration 0.1 mg/mL and living slices were incubated for 30 min in cisplatin-ACSF solution continuously saturated with a 95% O_2_-5% CO_2_ gas mixture at pH = 7.4. After that, slices were removed from the solution and immediately fixed for future electron microscopy (see Section 2.3).

### 2.3. Slice Preparation for Electron Microscopy

Slices were fixed in 2.5% glutaraldehyde, 4% paraformaldehyde in 90 mM sodium cacodylate buffer with 0.02 mM CaCl_2_ added and pH adjusted to 7.2–7.4. The slices were kept in the fixative at 5°C for 24 hours. After brief washing with 90 mM sodium cacodylate buffer, slices were washed in distilled water for 20 min. The slices were then dehydrated through a graded series of acetone and embedded in a 1 : 1 mixture of EMBed-812 and SPURR (EM Sciences). Ultrathin sections (50–60 nm) were cut with a Leica Ultracut Ultramicrotome, mounted on copper slot formvar-coated grids and examined with a transmission electron microscope JEM 100CX II (JEOL).

## 3. Results

### 3.1. Cisplatin Was Accumulated Mainly in Glial Cells of Rat Brain Slice, Mostly Adhered to Their Myelin and Ribosomes

**Figure 1:**
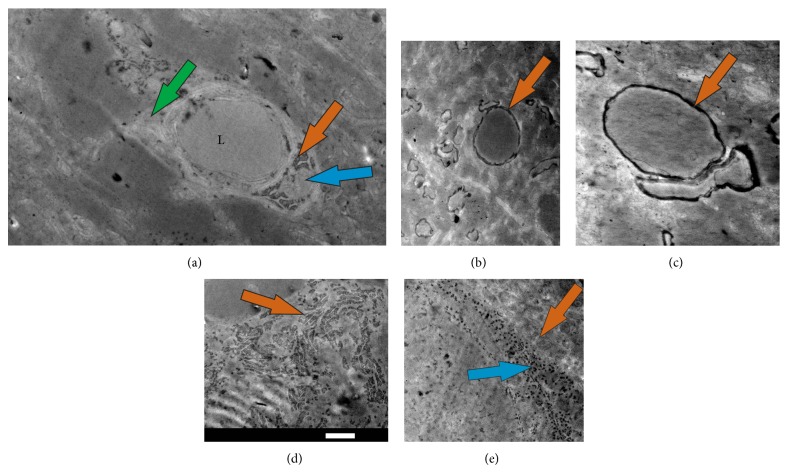
(a) Transverse cut of a small venule in the hippocampal area of a rat brain slice. The lumen of the blood vessel is marked by the letter “L.” A pericyte and specifically its endoplasmatic reticulum (orange arrow) that accumulated cisplatin are visible, while its nucleus is less stained (blue arrow). Also visible are astrocyte end-feet with mitochondria (green arrow). Membranes and mitochondria of endothelium cells are also visible. ((b) and (c)) Prominent accumulation of cisplatin in myelin of oligodendrocytes forming the sheath around nerve trunks in rat hippocampus. Orange arrows show the myelin, revealing that cisplatin probably reacts directly with some component of the myelin sheath, while there was no staining of nerve trunks themselves. (d) Rough endoplasmic reticulum in an astrocyte revealed by cisplatin. The orange arrow points to some ER cisternae, while the small black spots are the cisplatin-stained ribosomes. (e) Rough endoplasmic reticulum (orange arrow) and free ribosomes (blue arrow) in an astrocyte cell body (high magnification), showing ribosomes with adhered cisplatin. Scale: 1 micron for (a), (b), (c), and (d); 500 nM for (e).

We examined sections from rat hippocampus area for cisplatin staining. As we expected, cisplatin accumulation was found only in glial cells but not in neurons. Electron-dense material was visible in pericytes, astrocytes, and oligodendrocytes, recognized by their respective specific morphologies. Very prominent staining was found in the endoplasmatic reticulum (ER) of pericytes. A representative example of such staining of a pericyte situated adjacent to a blood vessel is shown in Figure 1. The prominently stained rough endoplasmic reticulum is the most visible element of this pericyte (Figure 1(a), orange arrow) while its nucleus is less stained (Figure 1(a), blue arrow). The nuclear region is found immediately adjacent to the endothelium, which is characteristic of pericytes [22]. Some sparse staining was also observed in the sheath around the blood vessel and pericyte (Figure 1(a), green arrow), most likely comprised of astrocytic end-feet.

The astrocyte end-foot contained visible mitochondria (Figure 1(a), green arrow) revealed by cisplatin staining, suggesting that cisplatin has some affinity for mitochondrial content as well as ribosomes.* Endothelial* cells also have some accumulation of cisplatin and their mitochondria and some membranes are also visible (Figure 1(a)).

*Oligodendrocytes* also displayed staining after acute cisplatin exposure; the most prominent accumulation of this drug was found in the myelin sheath around nerve trunks. Representative examples showing cisplatin staining of myelin around nerves are shown in Figures 1(b) and 1(c) (orange arrows). Interestingly, we observed no staining of the internal structure of these nerve trunks like in standard electron microscopy staining. In our preparations, the nerves look completely uniform. These data and the absence of clearly stained nerve cell bodies confirm that inside the brain the most sensitive elements accumulating cisplatin are glial cells.

*Astrocytes* can be easily recognized on preparations as they send their end-feet to blood vessels, and all other cells except for endothelial cells, pericytes, and oligodendrocytes appeared without specific staining by cisplatin. When we traced astrocyte cell bodies it appeared that the main cell element accumulating cisplatin was ER. An example of the rough endoplasmic reticulum in astrocyte is shown in Figure 1(d) (orange arrow), where the ribosomes are clearly visible. Interestingly, free ribosomes bind cisplatin even more effectively (Figure 1(e)), suggesting some elements of the ribosome are particularly adherent to cisplatin.

## 4. Discussion

The ability of the relatively heavy platinum atoms to disperse electrons makes them useful for electron microscopy, producing densely stained areas where platinum atoms are accumulated. Cisplatin is a well-known anticancer agent that contains platinum. Electron microscopy of cisplatin accumulation was previously employed to show a lack of vesicular transport of cisplatin in ovarian cancer cells [21]. We adapted this methodology to study cisplatin accumulation in living brain tissue after acutely applying cisplatin to hippocampal rat brain slices. Slices were maintained alive in cisplatin solution (0.1 mg/mL) for 30 min and then fixed and embedded in epoxy. No other heavy metals like osmium, uranium, or lead were used. We are confident therefore that only platinum (cisplatin) accumulation was revealed by electron microscopy without any other nonspecific staining.

It is known that cisplatin has neurotoxic side effects. While peripheral nerves are more vulnerable to cisplatin injury, some CNS damage, like cortical blindness, aphasia, and focal seizures are also present, especially when the drug is administered in higher doses [14, 15]. Neurotoxicity is, therefore, a significant factor affecting the efficacy of cisplatin treatment, as patients may experience even more negative side effects than benefits from this drug. Localization of possible cisplatin-mediated damage target, that is, specific affinity of cellular components to cisplatin in CNS, is important for assessing and predicting physiological level of its side effects and may give more comprehensive insight into the mechanisms cisplatin toxicity.

It has been established that cisplatin enters cells with the help of specific cation transporters, most notably OCTs and OCTNs [SLC22A1–5] [2, 3, 23–25]. The mechanism of uptake of cisplatin by OCTs may be similar to that of other polyamines, because cisplatin is the positively charged diamine (*cis*-diammineplatinum(II) dichloride). We have shown recently that OCTs are low affinity but high capacity carriers of di- and polyamines [26]. It was previously shown that OCTs and OCTNs transporters in mammalian CNS are overwhelmingly present mainly in glial cells [8, 9, 27]. In this study we also found that glial cells predominantly accumulated cisplatin in living brain slices, while neurons did not.

In pericytes (Figure 1(a)) and astrocytes (Figures 1(d) and 1(e)) cisplatin was accumulated in ribosomes: both ribosome associated with the rough endoplasmic reticulum (Figures 1(d) and 1(e)) and free ribosomes look completely dark because of the amount of bound cisplatin. Interestingly, the nuclei of these cells also had some moderate accumulation of the platinum drug, but the staining was significantly less pronounced (Figure 1). Our data support the suggestion by Heminger et al. [28] that RNA may be the main target of cisplatin. RNA constitutes the predominant material within the ribosome, which is approximately 60% RNA and 40% protein by weight [29], making the ribosomes extremely visible in electron microscopy after platination. Recently it was shown that accumulation of platinated adducts in total cellular RNA is 20 times stronger than in DNA [18]. Interestingly, accumulation of platinated DNA adducts was previously found by immunostaining in the mitochondria of cultured lung cancer cells and in the nucleus, preferentially at loci with high-density chromatin [30]. Mitochondrial injury was previously reported to be involved in cisplatin side effects [31]. Some but relatively mild staining was seen in the nucleus (Figures 1(a) and 1(e)) and in mitochondria (Figure 1(a)) of glial cells in our preparations.

The most visible electron-dense staining was present in oligodendrocytes, particularly in the myelin sheath surrounding axons (Figures 1(b) and 1(c)). Unlike pericytes and astrocytes, it was difficult to trace the cell body of these cells, but cisplatin-marked myelin sheaths were present in the majority of preparations, suggesting that myelin is one of the specific targets of this platinum-containing drug. Ultrastructural changes in myelin were not evident by electron microscopy after acute application of cisplatin in our experiment, but the high concentration of platinum product in the sheath suggests oligodendrocytes were the main target. The long term effect of the platinum drug directly on myelin is unclear. Myelin structure was unaltered in chemotherapy-induced neuropathy by cisplatin [32] and there is evidence that cisplatin can even reduce demyelination in autoimmune encephalomyelitis [33].

## 5. Conclusions

The* in vivo* “cisplatin staining” method validated that early targets of cisplatin in rat brain slices are myelin and ribosomes in glia.
